# The acceptability, feasibility, and usability of a virtual reality pain education and rehabilitation program for Veterans: a mixed-methods study

**DOI:** 10.3389/fpain.2025.1535915

**Published:** 2025-03-20

**Authors:** Amelia K. Mardon, Dianne Wilson, Hayley B. Leake, Daniel Harvie, Andre Andrade, K. Jane Chalmers, Aaron Bowes, G. Lorimer Moseley

**Affiliations:** ^1^ IIMPACT in Health, University of South Australia, Adelaide, SA, Australia; ^2^ The Pain Education Team to Advance Learning (PETAL) Collaboration; ^3^ NICM Health Research Institute, Western Sydney University, Westmead, NSW, Australia; ^4^ Quality Use of Medicine Research Centre, The University of South Australia, Adelaide, SA, Australia; ^5^ IPAR Rehabilitation, Melbourne, VIC, Australia

**Keywords:** chronic pain, Veterans, virtual reality, pain education, pain science education, acceptability, mixed methods

## Abstract

**Introduction:**

Persistent pain is a leading cause of medical discharges for Veterans. Pain science education (PSE) aims to better people's understanding about pain and is effective at reducing pain and depressive symptoms in Veterans. Preliminary evidence suggests virtual reality (VR)-delivered PSE has clinical benefits for people with persistent pain. This study investigated the acceptability, feasibility, and usability for VR-PSE for Veterans with persistent pain.

**Methods:**

Veterans (*n* = 7) and healthcare professionals (HCPs) experienced in treating Veterans (*n* = 5) participated in workshops that involved working through the VR-PSE program, online questionnaires, and a focus group. Quantitative data were analysed by descriptive statistics. Qualitative data were analysed using a framework analysis according to the Theoretical Framework of Acceptability (TFA). A mixed-methods analysis combined the quantitative and qualitative data via triangulation, with the findings presented according to the TFA domains.

**Results:**

The VR-PSE program was considered easy to use, engaging, and adaptable for different functional capabilities. Appropriate screening for contraindications prior to using the VR-PSE program was considered important by HCPs. Both Veterans and HCPs emphasized the need for a trusting client-clinician relationship to improve the acceptability of the VR-PSE program.

**Discussion:**

Overall, the VR-PSE program was found to be acceptable, feasible, and usable and may be a useful tool to incorporate into the clinical management of Veterans with persistent pain. Further research is needed to investigate the efficacy of VR-PSE programs on clinical outcomes for Veterans with persistent pain.

## 1 Introduction

Persistent pain is a pertinent problem among Veterans – it is a leading cause of discharges from the Defence Force due to medical reasons (1, 2) and it is more common in Veterans than the age-matched general population (3). Psychological comorbidities are common in war Veterans. Over 80% of Veterans are exposed to at least one traumatic event in their lifetime (4, 5) and over 30% of Australia's surviving male Korean War Veterans meet the criteria for post-traumatic stress disorder (6). It is important that clinicians adopt a trauma-informed approach when treating war Veterans to ensure interventions and healthcare services meet their unique needs.

Pain science education (PSE) aims to improve people's understanding about pain, including what pain is, how pain works, its dynamic multifactorial nature and protective function (7). When provided in tandem with other interventions, PSE is moderately effective at reducing pain and disability in people with chronic low back pain (8). A recent meta-analysis also concluded that pain education provides the most sustainable benefits in improving function and reducing fear of reinjury for people with chronic non-specific low back pain (9). Data specific to Veterans are limited but promising: a 6 month multifaceted intervention including “light touch” pain education intervention, in which all Australian Veterans prescribed opioids for non-cancer pain received written materials and an accompanying behavioural tool ['the Protectometer” (10)], avoided over 25,000 patient opioid months in the subsequent two years (11). Additionally, a small-sample pilot study suggested that pain education reduced both pain and depressive symptoms in Veterans (12).

Real-world data indicate that individuals who benefit from PSE are those who successfully achieve the learning outcomes (13). Additionally, insights from recovered consumers highlight the learning objectives that they perceive as most critical to their recovery (14). Analyses of randomised controlled trial data are also corroborative; a change in understanding of the problem seems to mediate the vast majority of clinical benefit of complex multimodal care for chronic back pain (13, 15). As such, the pain field has turned towards developing more effective education tools.

Virtual reality (VR) is an established tool to deliver effective education. Meta-analysis of 45 clinical trials showed that “immersive learning” improves attainment of learning objectives by up to 60% (16). Thus, VR presents a potentially powerful tool to improve learning, pain and disability outcomes in people with persistent pain. One VR-based pain education tool (Reality Health, Sydney, Australia) is being used by people with various persistent pain conditions in rehabilitation settings with promising results, including improved average pain and pain interference (17), and is feasible and acceptable to physiotherapists (18). However, the VR program was not specifically designed for Veterans challenged by persistent pain, nor has it been rigorously explored in this population. Considering the specific needs and context of Veterans, in particular with their likelihood of a history of trauma, it is important to formally establish the acceptability, usability, and feasibility, of the platform with Veterans before incorporating it into their care.

## 2 Materials and methods

### 2.1 Study design

This study used a convergent-parallel mixed-methods approach. That is, both quantitative and qualitative data were collected at the same time, analysed independently, and then integrated for interpretation. Ethical approval was sought from the Human Research Ethics Committee of the University of South Australia (no. 205079) and the Australian Departments of Defence and Veterans' Affairs Human Research Ethics Committee (DDVA HREC) (no. 482-23). The design and conduct of this study is reported in accordance with the Mixed Methods Article Reporting Standards (MMARS) (19). The study protocol was prospectively registered in Open Science Framework on 18 November 2023 (https://osf.io/gz5px/).

### 2.2 Participants and recruitment

Veterans and healthcare professionals (HCPs) were recruited for the study using purposive sampling. The inclusion criteria for Veterans were as follows: (i) previously engaged with eligible war service (i.e., operational service - participated in war-like operations outside Australia in areas where the level of risk is considered above that of normal peacetime conditions; continuous full-time service - served with one of the three branches of the Defence Force on a continuous full-time basis, as opposed to a part-time basis; and/or peacetime operations) or who were previously a member of the Defence Force (Navy, Army, Air Force); (ii) currently experiencing persistent pain (pain >3 months in duration). Health professionals were included if they: (i) held relevant tertiary qualifications in their clinical specialty (e.g., physiotherapy, medicine, psychology); (ii) had >2 years fulltime (or equivalent) experience treating Veterans with persistent pain; (iii) currently practicing clinically. All participants also had to have normal or corrected to normal vision, be proficient in reading, writing, and understanding English, and have access to the internet. Participants were excluded if they experience severe neurological symptoms with specific light patterns (e.g., photosensitive epilepsy).

Participants were recruited primarily by flyer advertisements at select Veteran services, as well as the professional network of the research team. We aimed, *a priori*, to recruit 15 participants for this study (10 Veterans and five HCPs) based on the recommended number of participants for focus groups (20).

### 2.3 Equipment and software

**Figure 1:**
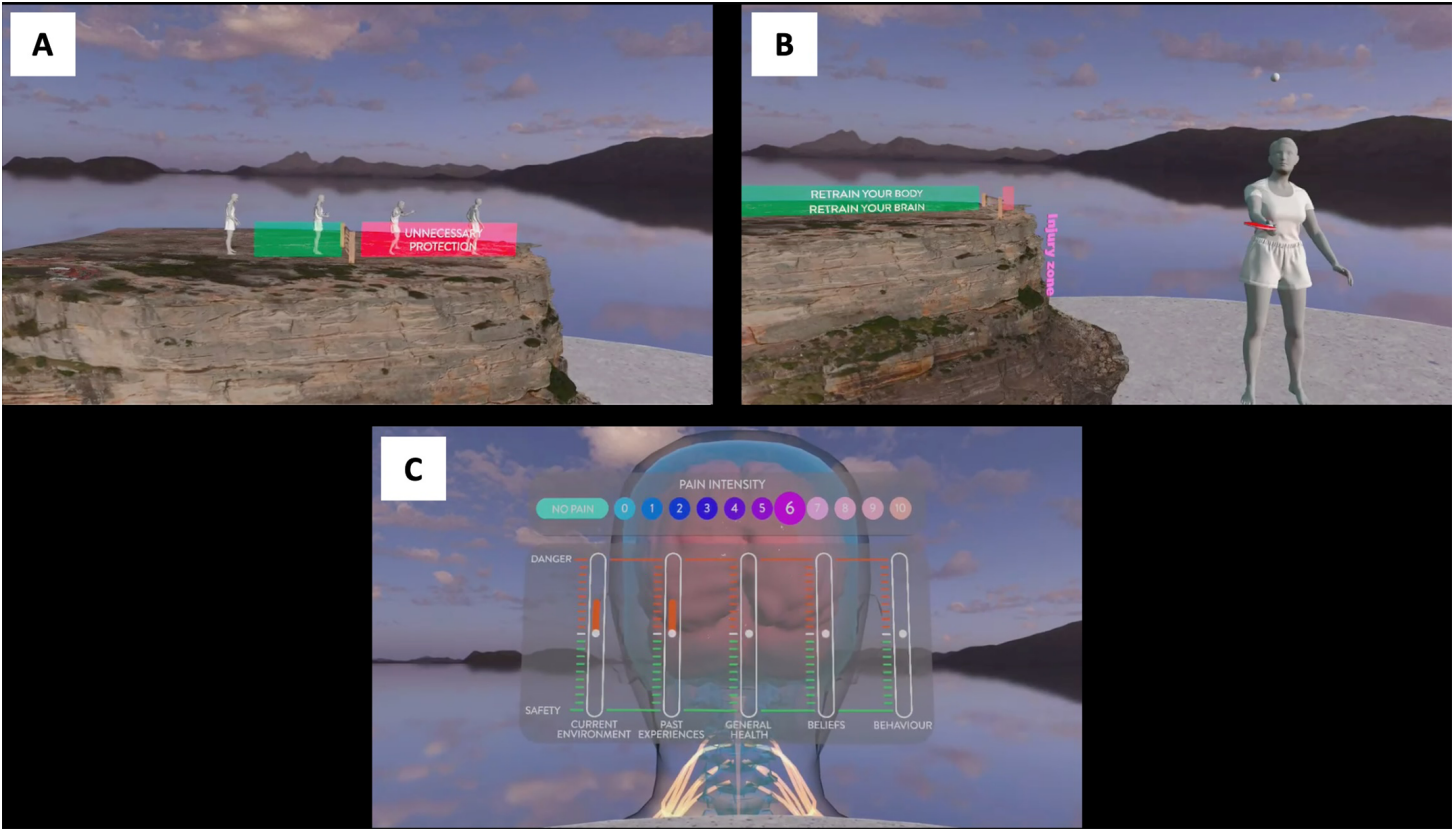
Visualisations of the VR-PSE program. **(A)** Understanding Pain. **(B)** Retrain your Body. **(C)** Retrain your Brain.

The virtual reality-based pain science education (VR-PSE) used a Meta Quest 2 head mounted display with connected touch controllers (Oculus, Facebook Technologies, LCC, Menlo Park, USA). The VR-PSE and rehabilitation program (Reality Health, Sydney, Australia) used in the study was designed to help educate people about pain and engage those with persistent pain in movement-based rehabilitation (Figure 1). The VR-PSE program included three PSE modules (M1: Understanding Pain; M2: Retrain your Body; M3a and b: Retrain Your Brain) and four movement-based rehabilitation modules (M4: Shapes; M5: Kites; M6: Lights; M7: Breathing). Further information about each module can be found in Supplementary File 1.

### 2.4 Data collection

An “inter-method” technique (21) was used for data collection prior to “mixing”. That is, qualitative and quantitative data were collected simultaneously using different methods. Quantitative data were collected using online surveys, whereas qualitative data were collected using focus groups.

#### 2.4.1 Eligibility screening and demographic information

Data obtained from the eligibility screening questionnaire for all participants included first name, email, age, sex assigned at birth, ethnicity, town and state of residence, primary language, previous experience with VR technology, and pain knowledge using the Revised Neurophysiology of Pain Questionnaire (rNPQ) (22). For HCPs, data were also collected on healthcare profession, tertiary qualifications, healthcare sector of work, and experience treating Veterans with persistent pain. For Veterans, data collected included Veteran status, level of education, history of persistent pain, pain duration, pain severity, and history of post-traumatic stress/post-traumatic stress disorder.

#### 2.4.2 Online survey

Following completion of the VR-PSE program, participants completed an online survey (hosted on Qualtrics), comprising of seven parts, to assess the acceptability, usability, and feasibility of the VR-PSE program.

Acceptability was assessed using the Theoretical Framework of Acceptability (TFA) questionnaire – a nine item tool developed according to the seven constructs relating to the acceptability of a healthcare intervention (affective attitude, burden, ethicality, intervention coherence, opportunity costs, perceived effectiveness, and self-efficacy) (23, 24). Items were rated using a five-point Likert Scale (strongly against – strongly for) and has demonstrated good discriminant content validity (23).

Feasibility was assessed using the Feasibility of Intervention Measure (FIM) (25) modified by the research team for VR. The FIM is a valid and reliable four-item questionnaire that evaluates the implementation of interventions into clinical practice (internal consistency (*α* = .89) (25). Items were rated using a five-point Likert Scale (completely disagree – completely agree).

Usability was assessed by three open-ended questions developed by the research team. The usability questionnaire allowed for quantifying how long participants took to get used to and complete the VR-PSE program, as well as any technical difficulties they experienced.

The extent to which participants experienced motion sickness related to the use of VR was assessed using the Simulator Sickness Questionnaire (SSQ) (26). The SSQ is a reliable 16-item questionnaire (internal consistency *α* = >.80) (26); symptoms (the 16 items) were rated on a zero to three scale (0 = not at all, 1 = mild, 2 = moderate, and 3 = severe). The score was then added across the 16 ratings, with a maximum score of 48.

The strength of VR embodiment was assessed using a modified version of an embodiment questionnaire, originally designed to quantify body ownership during the rubber hand illusion (27, 28). This four-item questionnaire evaluates the strength of VR illusory embodiment with a focus on participants' perceptions of VR avatar ownership (relating specifically to modules in which one's hands are visible as digitised hands within the virtual space). Participants rated their agreement with the four items using a three-level agreement scale (1 = disagree, 2 = somewhat agree, and 3 = strongly agree). The ratings were summed across the four ratings with a maximum score of 12.

Immersion within the VR environment was assessed using the valid and reliable Multimodal Presence Scale (internal consistency *α* = .84), specifically the five-item physical domain (29). Participants rated their agreement with each of the five items using a one to five Likert scale (1 = completely disagree, 2 = disagree agree, 3 = neither disagree nor agree, 4 = agree, 5 = strongly agree). Scores were summed across the five items with a maximum score of 25.

Pain knowledge was assessed using the rNPQ (internal consistency PSI = 0.84) (22) – a 13-item questionnaire where each item is answered either “true”, “false”, or “unsure”. Each question correctly answered counted one point to a maximum score of 13 points. This tool was selected because it does not include items that are directly addressed in the VR-PSE program and was thus thought to estimate generalizable or operationalizable knowledge shift.

### 2.5 Procedure

Potential participants completed an online eligibility screening questionnaire hosted on Qualtrics survey software (Provo, United States of America), which explained the nature of the study, sought electronic informed consent, and collected demographic data. Eligible volunteers were contacted by the primary researcher (AKM) via email to confirm availability and attendance at an in-person workshop.

A focus group guide was developed in accordance with the seven component constructs of the TFA (affective attitude, burden, perceived effectiveness, ethicality, intervention coherence, opportunity costs, and self-efficacy) (24) and a guide for developing focus group questions (30). This process involved developing questions based on the expertise and previous knowledge of the research team. Guidance was also taken from previous studies investigating the acceptability of VR interventions (31, 32).

Two four-hour workshops were held in Townsville, Queensland, Australia and facilitated by two members of the research team (DW and AB). Workshop 1 was conducted with the HCPs at IPAR Rehabilitation offices. Workshop 2 was conducted with the Veterans and held at Oasis Townsville, a Veteran-dedicated community facility. All participants attended their respective workshop at the same time. During each workshop, participants were instructed on how to use the VR headset and guided through the VR-PSE program and rehabilitation exercises (approximately 2.5 h in duration). Participants completed the program individually alongside the other group members. The workshop facilitators monitored participants throughout and provided assistance as required. The facilitators also aimed to reduce the amount of interaction between participants whilst they underwent the program (e.g., discussion, physical contact). Following completion of the VR-PSE program, participants completed the online survey assessing the acceptability, feasibility, and usability of the program. A focus group facilitated by researcher DW was then held, allowing participants to provide feedback on the VR-PSE program (one hour in duration). The focus group guide was developed prior to the workshop and the questions in line with the seven constructs of the TFA (affective attitude, burden, perceived effectiveness, ethicality, intervention coherence, opportunity costs, and self-efficacy) (24). Questions regarding the feasibility and usability of the VR-PSE program were also included in the focus group (see Supplementary File 2 for the focus group guide). The focus groups were audio recorded, transcribed using Descript transcription software (version 33.1.1; San Francisco, California), and checked for completeness prior to analysis.

### 2.6 Data analysis

**Figure 2:**
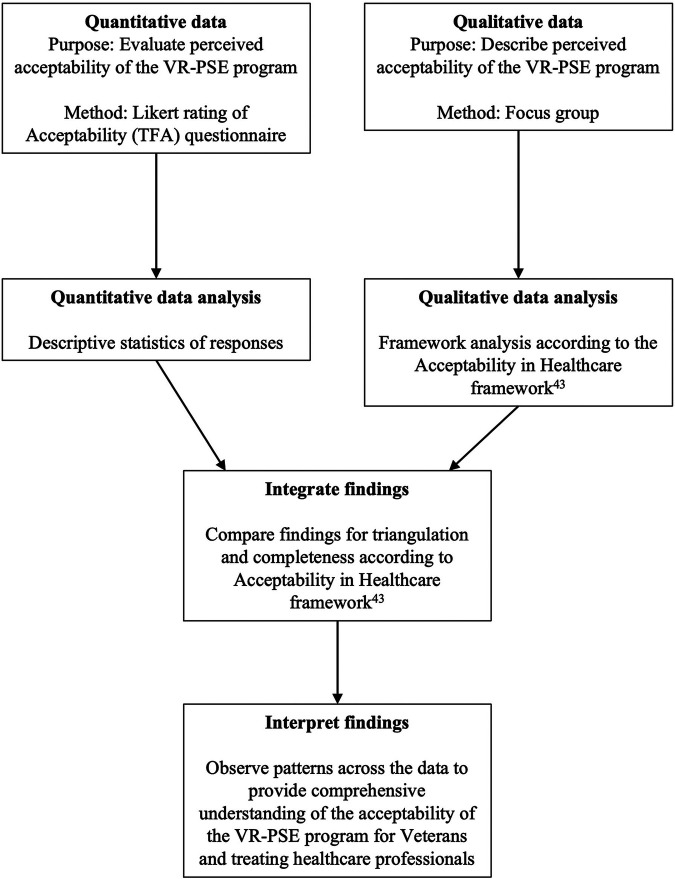
The convergent mixed-methods process. VR-PSE, virtual reality pain science education.

We took a convergent-parallel mixed-methods approach to data analysis (see Figure 2). Due to the nature of mixed-methods research and the integration of both quantitative and qualitative data, multiple epistemological views were taken to the data (epistemological pluralism) (33, 34). Both a post-positivist and constructivism epistemology were employed. Specifically, the postpositivist view allowed for an objective and generalisable assessment of the VR-PSE program, with less focus on the participants' perception of the intervention (35). In addition, the constructivist approach captured the intersubjective understanding of participants' engagement with the VR-PSE program, considering multiple realities of Veterans and HCPs. Combining these two paradigms allowed for an in-depth investigation into the acceptability, feasibility, and usability of the VR-PSE program for Veterans with persistent pain.

#### 2.6.1 Quantitative data

Quantitative data were exported from Qualtrics to Microsoft Excel. Demographic data and the distribution of Likert scale questions were analysed using descriptive statistics in IBM SPSS v26 (IMB Corp, Armonk, NY, USA) and presented as frequencies in a table. Change in pain knowledge for Veterans and HCPs was calculated using a repeated-measures t-test. Alpha was set at 0.05.

#### 2.6.2 Qualitative data

Qualitative data derived from the focus group transcripts were analysed using a framework analysis (36). A framework analysis was chosen because it emphasises both predefined constructs and emergent data when developing themes. In this study, data were primarily analysed according to the seven constructs of the TFA, while concurrently remaining open to discover new ideas and themes regarding acceptability, feasibility, and usability of the VR-PSE program.

In a series of steps, the primary researcher (AKM) first became familiar with the data by listening to the audio recordings and reading the transcripts multiple times. The data were then coded using a recursive, inductive approach, where codes were returned to and revised throughout the analytical process. Both the semantic (explicit) and latent (implicit) features of the data were considered throughout the coding process. Codes were then grouped deductively into preliminary themes according to the TFA constructs in a framework matrix (24). Preliminary themes were iteratively reviewed and refined with the research team to determine final themes prior to “mixing” with quantitative data. Representative quotes were selected to illustrate themes.

#### 2.6.3 Integration

Central to the effectiveness of a mixed-methods study is integration of both quantitative and qualitative data to draw insights beyond the information gained from the separate results alone. In this study, the rationale for “mixing” quantitative and qualitative data was for triangulation and completeness. Triangulation allows for multiple methods with offsetting biases to assess the same phenomenon (37). Thus, triangulation allowed for the integration of quantitative (online survey) and qualitative (focus group) data, both related to the acceptability of a VR-PSE program for Veterans with persistent pain. That integration reveals the extent of convergence and corroboration allowing confidence in the validity of the findings (37). Integrating quantitative and qualitative data also allowed for a more comprehensive account of the feasibility, acceptability, and usability of the VR-PSE program (i.e., its completeness) (21). Data integration occurred after the independent analysis and interpretation of the quantitative and qualitative data.

## 3 Results

We recruited seven Veterans and five HCPs for this study. This was a deviation from our protocol; due to time constraints with recruitment we were unable to reach our target sample size for the Veterans. Veterans' demographics are presented in Table 1 and HCPs' demographics presented in Table 2.

**Table 1:** Veteran demographics.

Participant ID	Age	Sex assigned at birth	Time since last deployment (years)	Pain severity most days	Location of pain	Duration of pain	Experience of PTS/PTSD	Confidence using VR	rNPQ score (pre-workshop)
V1	51–60	Male	5	Moderate (4–6)	Back, hip	4–6 years	No	Not at all confident	11
V2	41–50	Female	14	Mild (0–3)	Right hip, lower back	10+ years	No	Unsure	10
V3	51–60	Male	16	Severe (7–10)	Knees, legs	1–3 years	Yes	Somewhat confident	1
V4	71+	Male	53	Severe (7–10)	Head, neck, shoulders, back, hips, lower legs, and feet.	10+ years	Yes	Unsure	3
V5	31–40	Male	N/A	Moderate (4–6)	Back, neck, hips, knees, ankles, shoulders	7–9 years	Yes	Neither confident nor unconfident	0
V6	41–50	Male	21	Severe (7–10)	Lower back, feet	10+ years	No	Somewhat confident	2
V7	41–50	Male	5	Moderate (4–6)	Back, neck	6–12 months	Yes	Not at all confident	6

PTS/PTSD, post traumatic stress/post traumatic stress disorder; rNPQ, revised neurophysiology of pain questionnaire; VR, virtual reality.

**Table 2:** Healthcare professionals’ demographics.

Participant ID	Age	Sex assigned at birth	Clinical specialty	Healthcare sector	Experience treating Veterans with persistent pain	Experience with technology-based health interventions	Experience with VR interventions	rNPQ score (pre-workshop)
HCP1	51–60	Female	Nurse	Public, Private	6–10 years	No	No	12
HCP2	31–40	Male	Physical therapist	Public	6–10 years	Yes	No	13
HCP3	51–60	Male	Medical doctor	Public, Private	10+ years	Yes	No	10
HCP4	18–30	Female	Nurse	Public, Private	2–5 years	No	No	11
HCP5	41–50	Male	Occupational therapist	Public, Private	10+ years	Yes	No	13

rNPQ, revised neurophysiology of pain questionnaire; VR, virtual reality.

### 3.1 Quantitative analysis

Ratings for the quantitative survey are displayed in Table 3. Overall, both Veterans and HCPs rated the VR-PSE program highly on the TFA questionnaire and FIM. Veterans and HCPs scored low on the SSQ. For the embodiment questionnaire and Multimodal Presence Scale, Veterans and HCPs scored highly. For “usability”, Veterans reported no interruptions with the VR-PSE program. HCPs recorded multiple minor technology issues, which included: (1) hand/controller tracking disruptions (*n* = 1), (2) technical interruptions (*n* = 2), (3) issues with adjusting headset while wearing glasses (*n* = 1), and (4) disruptions with other participants in the room (*n* = 1).

**Table 3:** Quantitative survey measures post-workshop.

Outcome measure	Veterans *N* = 7 Median (range)	HCPs *N* = 5 Median (range)
TFA score (1–45)	38 (35–45)	38 (34–40)
FIM score (1–20)	17 (15–20)	16 (15–19)
SSQ score (0–48)	1 (0–13)	5 (2–10)
Embodiment questionnaire score (1–12)	9 (7–12)	10 (8–12)
Multimodal Presence Scale score (1–25)	22 (16–25)	19 (17–24)
rNPQ score (pre-workshop; 0–13)	3 (0–11)	12 (10–13)
rNPQ score (post-workshop; 0–13)	8 (5–11)	12 (9–13)
Length of time taken to complete VR program	2–2.5 h	0.75–2 h
Length of time taken to feel comfortable using VR	0–5 min	0–10 min

TFA, theoretical framework of acceptability; FIM, Feasibility of Intervention Measure; SSQ, simulator sickness questionnaire; VR, virtual reality; rNPQ, revised Neurophysiology of Pain Questionnaire.

### 3.2 Qualitative analysis

Veterans and HCPs provided accounts of their experiences engaging with the VR-PSE program. The themes reflect the seven constructs of the TFA, including: (1) affective attitude; (2) burden; (3) ethicality; (4) intervention coherence; (5) opportunity costs; (6) perceived effectiveness; and (7) self-efficacy.

#### 3.2.1 Affective attitude

Affective attitude refers to how Veterans and HCPs feel about the VR-PSE program. Overall, both Veterans and HCPs reported that the program was a novel and engaging way of learning about pain. Veterans also emphasised the need for the VR-PSE program to be introduced early in their pain rehabilitation journey.

##### 3.2.1.1 A novel way of learning

All Veterans and HCPs described the VR-PSE program as being an engaging, interactive, and fun method of learning about pain. One Veteran described how they were able to take in more information using VR than if they were given the information by a presentation where you “*just tune out”* (Veteran). Similarly, some HCPs noted that because VR is “*still relatively new”* that it would be a fun alternative way to learn about pain.

“It’s quite fun, you know, something a bit different, you are not coming into a room and just getting, you know, talked at by like a lecturer sort of thing.” - HCP

Some participants suggested that the VR-PSE program was engaging because of how immersive the virtual environment was. For example, a healthcare professional reflected on their multi-sensory experience when going through the education modules:

“I think the things that were supposed to be quite real, felt quite real. Like the cliff, I was looking around the waves were crashing, the wind, like I could hear the wind, the fire was really good for me, I got tingles in my hands from being too close to the fire, so that was really cool.” - HCP

##### 3.2.1.2 Education necessary early in pain journey

While Veterans, generally, described the VR-PSE program as being acceptable for their pain rehabilitation, they emphasised that the education should be introduced earlier in their pain journey – that “*it's too late at this point”* (Veteran)*.* One Veteran described how it may be of more benefit to introduce pain education during Defence members' training. In the face of injury, Defence personnel could then implement the learnings and potentially improve their clinical outcomes.

“Maybe take another step back, so instead of the Soldier Recovery... moving [the education] forward to your IET where you do your Initial Employment Training, and they incorporate it there. So you’re capturing the soldiers that have done their initial say, six months, they’ve gone to the next stage of their career… So if there is an injury from then on, they’ve got an understanding from a pain side, the understanding, this is what’s happening, this is how I can assist me with my career and pain. Which in the long term is going to save Defence money.” - Veteran

#### 3.2.2 Burden

Burden relates to how much effort Veterans and HCPs perceive to be required to engage with the VR-PSE program. Veterans described how the VR-PSE program was less burdensome than anticipated because it was accessible for different functional capabilities. HCPs highlighted some technical errors that required effort to rectify.

##### 3.2.2.1 Accessible for different functional capabilities

Several Veterans expressed their appreciation of the VR-PSE program being accessible for people with varied functional capabilities. However, Veterans applauded that the VR-PSE program could be done sitting or standing and depending on their capabilities.

“Can I give you a positive here? So even in this room, you can do it standing up or even if you’re a wheelchair or you need to sit down, you can still achieve. So it’s catering for multiple people. So that’s a positive.” - Veteran

That the VR-PSE program was accessible for varied capabilities was emphasised by one Veteran who completed the program sitting down. He stated that the program was “*fine, yeah manageable”* (Veteran) and appreciated that there were activities in which he could engage without having to “*get up and run around and chase something”* (Veteran). One healthcare professional reflected on a potential risk of using VR with Veterans, stating that it “*could potentially flare symptoms”* (HCP).

##### 3.2.2.2 Technology glitches

Several HCPs detailed issues with the VR technology, which was a common reason for the burden of engaging with the intervention. Technology glitches included losing the visualization of their hands in the VR environment and losing their space parameters. These interferences were a cause for unnecessary time spent on the program, a potential barrier to its use given the short appointment times HCPs have with clients. For example:

“There was obviously your controllers for some reason… I need to spend ten minutes to turn it on and turn it off again.” (HCP)

The Veterans underwent the same process to engage with the VR-PSE but did not report any of the technical difficulties reported by HCPs. The disparity between the two groups in this aspect of the tool may relate to the group members, the location in which testing occurred, the day on which the testing occurred, or a combination of the above.

#### 3.2.3 Ethicality

Ethicality refers to how well the VR-PSE program fits with the values of Veterans and HCPs. Both Veterans and HCPs emphasised the importance of trust between the client and treating HCP when delivering such an intervention. HCPs also voiced the value of appropriate screening prior to implementing the VR-PSE program with Veterans.

##### 3.2.3.1 Trust

All participants described the importance of trust and good patient-clinician relationships in persistent pain management for Veterans. From the HCPs, they said Veterans would require trust and rapport with the clinician to “*feel much better”* (HCP) knowing they were there to provide support.

Similarly, and tying together with the TFA construct “perceived effectiveness”, one Veteran reported that trust toward the healthcare professional would be a key factor in the successful implementation of the VR-PSE program. They suggested that previous failed attempts at pain management, coupled with the cynical nature of Veterans, would be barriers for engaging with the VR-PSE program; these barriers could be alleviated with a strong patient-clinician relationship.

“I think the effectiveness of it, for Veterans, especially older Veterans who have had pain for such a long period of time, I think the success would come with trust. The individual needs to trust the practitioner that is leading this. If they don’t have that, then you’re not going to get buy in. Because people are quite cynical about different treatment methods, we are all like that. And unfortunately, Defence members tend to be the worst because we don’t want any of that hippy, happy, clappy shit around us… so I do think it is about trust and because we are talking about pain, that is debilitating and is with us all the time, that yeah. So if you can figure out how to try and build that trust and that rapport, you’ll have a better success rate, especially with Veterans.” - Veteran

##### 3.2.3.2 Appropriate screening

All HCPs noted the importance of appropriate screening of the Veterans prior to delivering the VR-PSE program in clinical practice. The most common condition for which to screen was post-traumatic stress disorder. For example, one HCP stated that the VR headset may be a trigger for Veterans, particularly for those with “*an experience of night vision goggles and contact”* (HCP).

One HCP also highlighted that one of the virtual locations featured in the VR-PSE program was a common suicide location for those who lived in that general area. Given Veterans experience higher rates of suicide than the general population (38), the HCP participant expressed that without appropriate screening or forewarning, this part of the VR-PSE program may not be suitable for some Veterans.

“Yeah, I mean look there would be some guys that would recognise where that is like that particular area I think is a common suicide location so having that in, I actually think some would respond angrily but again like HCP3 said maybe prerequisite is stable PTSD.” - HCP

#### 3.2.4 Intervention coherence

Intervention coherence describes the extent to which Veterans and HCPs understand the VR-PSE program and how it works. Veterans suggested that one way to improve the coherence of the program would be to include Veteran specific learning modules, including the voices of Veterans with lived experience.

##### 3.2.4.1 Veteran specific content

At face value the VR-PSE program was received very well by Veterans, but several suggested that the intervention could be enhanced by adding content specific to Veterans. For example, one Veteran suggested rehabilitation modules specifically for knee, ankle, and hip pain because they are common among the cohort (of note, the Reality Health platform does include knee modules, but they were not used in this study). Incorporating content specific to Veterans was said to be of value because they “*have different experiences”* (Veteran). The addition of lived experience voices throughout the VR-PSE program was also recommended by one Veteran to improve engagement.

#### 3.2.5 Opportunity costs

Opportunity costs refer to the benefits or values that must be given up by Veterans and HCPs to engage with the VR-PSE program in clinical practice. Veterans detailed how the “cost” of VR for pain rehabilitation may not be so extensive because it is already being used by the Defence Force for training. However, Veterans also expressed that acceptability may be inhibited because the learnings do not align with what they see as the Defence Force approach to pain management.

##### 3.2.5.1 VR already in use by Defence

Some of the Veterans explained how VR is currently being used in training programs for Defence Force personnel. Because of this, Veterans would be accustomed to using VR technology and wouldn't require extensive training on how to use the equipment. One Veteran used the example of VR firefighting training currently being delivered by the Defence Force.

“Vets are going to VR now with their training… they’re doing VR firefighting now… so you see them learning and putting a tire fire out or something like that… so it’s the way the world’s going.” - Veteran

The Veteran also expressed that if the Defence Force was to integrate the VR-PSE program into their initial training that there wouldn't be any additional “*cost and the time”* (Veteran) lost because they already have the infrastructure to use it.

##### 3.2.5.2 Education learnings go against common Veteran mindset

Many Veterans described that the learnings within the VR-PSE program go against the mentality and values generally held by Veterans. While Veterans in this study emphasised that the education was useful, they also warned that it may be difficult to get buy-in from Veterans in general because the messaging is counterintuitive to what is endorsed in the Defence Force. For example, one of the Veterans described how he was forced to return to high-intensity work despite being injured because that was the norm.

“But the end point is, if you’ve got bosses who’ve never been injured, and then a soldier comes in, I was on crutches when I come back to work and I was expected to pick up exactly where I was left off. And I tried not to, but in the end I had no choice.” - Veteran

To counteract this problem, some Veterans suggested that the VR-PSE program be used to educate everyone in the Defence Force about persistent pain. Specifically, Veterans said pain education needs to start “*at the top, the hierarchy”* because “*if they’re not aware of it, [integrating the pain education learnings] is not going to happen”* (Veteran).

#### 3.2.6 Perceived effectiveness

Perceived effectiveness describes whether Veterans and HCPs perceive the VR-PSE program to achieve its purpose. Veterans and HCPs iterated that the VR-PSE program would be effective for use by Veterans with persistent pain, in particular for a graded-learning approach alongside other pain management strategies.

##### 3.2.6.1 A key piece of the multidisciplinary puzzle

All of the HCPs described that the VR-PSE program would be an effective pain education tool because of its engaging and interactive nature; that it would “*[help] solidify a lot of that, some of those foreign concepts we try to get across to the patients”* (HCP). HCPs suggested that the program could be improved with incorporating revision questions to test and solidify learnings.

Some of the HCPs stated that the VR-PSE program would be effective when used in conjunction with other pain management strategies. For example, one HCP highlighted that the rehabilitation modules will be useful for increasing Veterans' confidence to engage in movement.

“The activities would help definitely I think, make them more able to do things and then more, it will stop them from saying they can’t when obviously they can because we’ll have videoed them doing it and you can say, ‘Well this is you doing it, so you can’.” HCP

The notion of incorporating the VR-PSE program as part of a multidisciplinary management approach was advocated by Veterans saying it was good for learning but not for “fixing” their pain. One Veteran described how other HCPs and pain management strategies can then be used to build upon the education content.

“The thing I learnt today is it doesn’t matter how you look at it, it’s just an information session… there’s nothing to help you fix something… so you need to go from the information session and then, other people need to build on that.” - Veteran

#### 3.2.7 Self-efficacy

Self-efficacy relates to Veterans' and HCPs' confidence that they can perform the behaviors to engage with the VR-PSE program. Generally, Veterans and HCPs reported that the VR-PSE program was easy to use but training and instructions were important for successful implementation.

##### 3.2.7.1 Easy to use

All of the Veterans expressed that the VR-PSE program was intuitive and easy to use. Regarding the PSE content specifically, some of the Veterans said that the content was “*very easy to understand”* (Veteran) because it used “*simple language”* (Veteran). One Veteran described his enthusiasm for the VR-PSE program despite having very low confidence in using VR technology prior to the workshop:

“The things with me, technology I hate it like you wouldn’t believe it. It’s the devil for me, I hate it. And technology hates me a lot as well, and with this thing it was easy to understand, easy to operate, a bit slow but for me, not liking technology it was good. I actually enjoyed it and the information session was good. And it was almost like a one to one to be honest.” - Veteran

##### 3.2.7.2 Training and instructions to boost self-efficacy

Both Veterans and HCPs emphasised that training and clear instructions on how to use the VR-PSE program is important for ensuring confidence and self-efficacy while using the program. One HCP recommended that the activities shouldn't “*commence until you have really listened to your instructions properly”* (HCP) because you may not learn the education content nor understand the activity's purpose.

Another HCP also warned that progressing with activities without listening to instructions may exacerbate symptoms for Veterans with persistent pain.

“Like I, probably it’s my fault, but I just went, yeah, I know what I’m doing and off I went, and I started doing stuff and it let me start doing stuff without actually listening to the instructions… But it maybe shouldn’t let you do stuff until it’s finished doing the instructions because you are, you can probably do yourself a bit of an injury if you are a bit sensitive.” - HCP

Most of the Veterans agreed with the HCPs, that it was beneficial to receive instructions prior to and during the VR-PSE program. For example, one Veteran described that being informed of potentially triggering components was helpful for reassurance and preparedness.

“And you frontloaded us with a lot of information before we went in and actually did the program… I think it was good just being aware that, yeah you’re going to be coming up to a cliff and it’s only a couple of seconds.” - Veteran

However, one Veteran stated that too much information may take away from the excitement and engagement of the program. Appropriate screening by HCPs prior to using the VR-PSE program in clinical practice may assist with determining a suitable amount of information each Veteran requires to get the most benefit.

“On the other side of the coin, if you don’t front load us with a lot of it, like from me sitting here watching you all go around, when you clap, then the next minute you’re on a cliff… you could see the different people, how they’re reacting, so it was, I think if you don’t front load us, it gives you better value for money. Because I knew that as soon as I clap, the cliff was coming. I know what you’re saying, but some of the unknown helps in other ways.” - Veteran

### 3.3 Integration

This study integrated quantitative and qualitative data for triangulation and completeness. Table 4 presents a joint display of quantitative findings with sub-themes and representative quotes from the qualitative framework analysis to provide an overall understanding of the of the entire data set. The TFA constructs that were complemented by the two data sets included “affective attitude”, “burden”, “ethicality”, “perceived effectiveness”, and “intervention coherence”. For example, clinicians reported technical issues with the VR headset whereas Veterans did not report any difficulties. These qualitative findings were supported by the TFA questionnaire – the median rating for the ethicality item was lower for HCPs than Veterans. There was divergence between quantitative and qualitative data for the TFA constructs “self-efficacy” and “opportunity costs”. The framework analysis revealed that Veterans and HCPs felt self-efficacious to use the VR-PSE program. However, meta-inferences inferred that there was a discrepancy between the two datasets; participants perhaps emphasizing the need for further instructions embedded in the program in the quantitative survey more so than the focus group where they described that the program was intuitive and easy to use.

**Table 4:** Integration of quantitative and qualitative findings.

TFA construct TFA questionnaire item	Quantitative finding (median Likert rating; range) (0 = strongly against; 5 = strongly for)	Qualitative excerpt	Meta-inferences’ for triangulation and completeness
Affective attitude *Did you like or dislike the VR program?*	5 (4–5)	“It's quite fun, you know, something a bit different, you are not coming into a room and just getting, you know, talked at by like a lecturer sort of thing.” (HCP) “For me, not liking technology it was good. I actually enjoyed it and the information session was good. And it was almost like a one to one to be honest.” (Veteran)	VR-PSE program was fun, interactive, and engaging.
Burden *How much effort did it take to use the VR program?*	4 (4–5)	“Yeah, my hands disappeared for a small amount of time.” (HCP) “The things with me, technology I hate it like you wouldn't believe it. It's the devil for me, I hate it. And technology hates me a lot as well, and with this thing it was easy to understand, easy to operate.” (Veteran)	Minimal effort required to use the VR-PSE program. HCPs did report some minor technical difficulties.
Ethicality *How fair is the VR program for Veterans to use with chronic pain?*	4.5 (4–5)	“HCP: I think you made a good point before about the headsets being on people, they might get a bit of claustrophobia. HCP: Well particularly if they’ve had an experience of night vision goggles and contact. HCP: Yeah, so having screening around that would be really helpful.” (HCPs) “You can do it standing up or even if you’re a wheelchair or you need to sit down, you can still achieve. So it's catering for multiple people. So that's a positive.” (Veteran)	Appropriate screening is required to ensure the VR-PSE program is fair to use for Veterans with chronic pain.
Perceived effectiveness *The VR program will help Veterans understand their pain.*	4 (4–5)	“The education that is being provided and I think the multifactorial implementation with different diagrams and models was really quite immersive and helped solidify a lot of that, some of those foreign concepts we try to get across to the patients.” (HCP) Interviewer: “So it was useful for you because it reinforced what you already knew, but you would have like it earlier in your journey?” Participant: “Yeah definitely.” (Veteran)	The VR-PSE program provides a novel way for Veterans with persistent pain to better understand their pain.
Intervention coherence *It is clear to me how the VR program with help Veterans learn about their pain.*	4.5 (4–5)	“Are you looking at having more rehab programs developed?.. Knees… Yeah because they’re the big ones. Even ankles as well, is another big one. Yeah.” (Veteran)	“Veteran-specific” modules would help improve coherence and relatability of the VR-PSE program.
Self-efficacy *How confident did you feel about using the VR program?*	4 (1–5)	“[Veterans] might like knowing like how to recalibrate where you are in space relative to the oculus ahead of time… so they can just do it themselves.” (HCP) “So it was easy to use, it was easy to understand and it was easy to listen to.” (Veteran)	Ensuring Veterans and HCPs are appropriately trained and instructed on how to use the program is important for feeling confident while using the VR-PSE program.
Opportunity costs *Using the VR program will interfere with my other priorities.*	4 (1–5)	“In training, and even soldier recovery level, they’re using VR for different applications” (Veteran)	Opportunity costs may be low because VR is already used in Defence training and thus they are already familiar with the technology.

## 4 Discussion

This mixed-methods study found that a VR-PSE and rehabilitation program is acceptable, feasible, and usable by Veterans with persistent pain and their treating clinicians. In line with the TFA constructs, the VR-PSE program was acceptable because of its easy use (“self-efficacy”), novelty (“affective attitude”), and ability to be used by Veterans with varying functional capabilities (“ethicality”). Integration of the framework analysis and the quantitative ratings from the TFA questionnaire highlights convergence and divergence between Veterans' and HCPs' perspectives on the VR-PSE program. We discuss in greater detail considerations for implementing the VR-PSE program in clinical practice for Veterans with persistent pain.

Virtual reality appears to be a novel and engaging method for delivering PSE to Veterans with persistent pain. Consumers have described that PSE is hard to take on board (39, 40). The use of VR may reduce this barrier given the VR-PSE program was well received by the Veterans in this study, who describe themselves as being particularly hard to please regarding their pain management. The findings of this study add to the preliminary real-world data from occupational rehabilitation settings that VR is an effective tool to deliver pain education for people with persistent pain (41) with the potential to improve clinical outcomes (17). Clinical benefits, such as reduced pain and disability, from a VR-PSE program are likely to be mediated by the enhanced reconceptualization of pain, although we did not set out to test this idea and our design would not allow it. Certainly, Veterans displayed an increase in pain knowledge following the workshop. This may be due to the achievement of specific learning objectives [initially identified by recovered consumers to be most helpful for their recovery (14)] through a VR-facilitated immersive embodied experience (17). The use of VR to deliver pain education may also reduce barriers commonly reported by HCPs, including difficulty delivering “good” pain education within short appointment times (42). The VR-PSE program is currently being used by consumers both in the clinic and at home under the guidance of an HCP. The ability of VR to be used at home may improve accessibility of PSE. However, the costs associated with VR technology should be considered, including set-up costs for the HCP and out of pocket costs for consumers.

Veterans emphasised the importance of integrating their lived experience into the VR-PSE program. Specifically, they wanted content on pain conditions that are common among Veterans, including hip and knee pain. This request is consistent with the theoretical frameworks that guide contemporary approaches to the PSE, most obviously constructivism (41). In this framework, people learn through actively constructing their knowledge based on their own experiences and are active in their learning rather than just a passive recipient of information (43). The integration of consumer perspectives and context within education material is at the crux of constructivism (41). This approach aims to counteract one key barrier to the uptake of traditional pain education interventions - that the content is not applicable nor relevant to the consumer, and therefore perceived as not being useful or beneficial (40). While Veterans in this study did describe the VR-PSE program as useful and engaging, it may be enhanced by including specific content to Veterans' experiences. Given this program has been developed for multiple one-on-one sessions under the guidance of a HCP, VR may be a preferable but not necessary tool to deliver any Veteran-specific content. Additional learning materials that are more cost-efficient, such as web resources or workbooks, may be appropriate alternatives to provide alongside VR-PSE. This may overcome limitations reported here without incurring excessive development costs.

Beyond the content, it is also important to consider how pain education should be best delivered. One motivator for this study was to explore whether VR is considered safe for use by Veterans given they are more likely to experience PTSD and traumatic injuries than the general population is (38). These specific contexts are important to consider when developing and delivering interventions for Veterans with persistent pain, including education. While the HCPs in this study raised concerns about the VR-PSE program to be a potential PTSD trigger, the Veterans did not voice the same concerns, despite many of them reporting a history of PTSD. These findings are in line with preliminary evidence that VR technology appears beneficial for improving PTSD symptoms across different populations (44). Similarly, the HCPs queried whether the VR program would be appropriate for use by Veterans with functional limitations, such as upper limb function. This study found that Veterans of various functional capabilities were able to successfully complete the VR-PSE program, despite not be initially developed to cater to those needs.

This study supports the need for a whole of community approach to PSE. While Veterans in this study described how PSE should be introduced earlier into the Defence Force training, they also emphasized that this would be difficult to achieve because the learnings of the VR-PSE program conflict with their experiences of how pain is managed in the Defence Force. Community level pain education, including upskilling of HCPs working with people with persistent pain, is one approach being taken to tackle the widespread misconceptions about pain (45). For the Veteran community specifically, pain education has been integrated into initiatives such as the Veterans' Medicines Advice and Therapeutics Education Services (MATES) programme with a good effect on opioid prescriptions (11). Similarly, the United States Veterans Health Administration endorse the Stepped-Care Model of Pain Management (46) – a three-tier, holistic approach for Veterans with persistent pain that involves the continual development of Interdisciplinary Pain Management Centres and places pain education at the heart of intervention. These initiatives have demonstrated a positive impact on clinical outcomes for Veterans with persistent pain, including decreased opioid use (11).

Strengths of this study include: the use of a mixed-methods approach that allowed for an in-depth exploration into the acceptability, feasibility, and usability of a VR-PSE program for Veterans with persistent pain; inclusion of two key interest holders – Veterans with persistent pain and HCPs; *a priori* registration of the protocol and transparent reporting of deviations, as recommended for all pain research (47). The study also had limitations. First, the use of focus groups may reduce the richness of the qualitative data collected, as compared to one-on-one interviews. Second, our sample size was smaller than intended because we were limited to a small geographical location in Australia and had a short time frame to recruit and conduct the workshops. Although we targeted a region that has the largest Army base in Australia, it does limit the generalizability of our findings. Third, we did not assess equity-related characteristics across all relevant domains (48–50) – an oversight that also limits the generalizability of our findings. Last, due to the pragmatic nature of the study, the workshop did not replicate exactly how the VR-PSE program is intended for use in clinical practice, which is over several sessions on different days. Some HCPs did note that the workshop environment caused disruptions (e.g., too many participants in the one space) which would not occur in most clinical settings. Completing the program in its entirety in the one session may also have impacts on participant engagement and knowledge retention. Notably, this was not reported and did not seem to impact the experience overall with using the VR-PSE program. A final limitation of the current work is that its scope and constricts meant that we were not able to recruit participants with a wide range of chronic pain diagnoses and a wide range of pain intensity and impact levels. This should be considered when planning future clinical trials.

Future research may explore further tailoring PSE content to Veterans with persistent pain. Similar work has been performed for other pain populations, including pelvic pain (51), complex regional pain syndrome (52), and adolescent pain (53), and has been instrumental in developing targeted pain education curricula. A clinical trial to investigate the efficacy of the VR-PSE program in a larger sample of Veterans with persistent pain may be indicated given there is a paucity of data on VR-enhanced pain education for Veterans with various pain conditions and a history of trauma. Although extensive evidence supporting the clinical benefit of pain education (8), that its effects are mediated by learning certain target objectives (13), and that VR-based learning improves learning outcomes (16) should be considered in designing, and weighing up the cost-benefit, of a clinical trial.

## 5 Conclusion

This mixed-methods study found that a VR-PSE program was acceptable, feasible, and usable by Veterans with persistent pain and HCPs experienced in treating Veterans. The VR-PSE program was considered easy to use, engaging, and adaptable for different functional capabilities. Appropriate screening for contraindications (e.g., PTSD) was considered important by HCPs, and both Veterans and HCPs emphasized the need for a trusting client-clinician relationship. Our sample size was smaller than expected. This raises two important considerations. First, it suggests that, although participants were very positive about the VR-PSE once they tried it, there may be barriers to overcome for Veterans to consider trying it. Second, the current feasibility findings would be strengthened by replication in a larger sample.

## Data Availability

The raw data supporting the conclusions of this article will be made available by the authors, without undue reservation.

## References

[1] Gauntlett-Gilbert J Wilson S. Veterans and chronic pain. Br J Pain. (2013) 7(2):79–84. 10.1177/2049463713482082 26516504 PMC4590126

[2] Gatchel RJ McGeary DD Peterson A Moore M LeRoy K Isler WC Preliminary findings of a randomized controlled trial of an interdisciplinary military pain program. Mil Med. (2009) 174(3):270–7. 10.7205/MILMED-D-03-1607 19354091

[3] O’Toole BI Catts SV Outram S Pierse KR Cockburn J. The physical and mental health of Australian Vietnam Veterans 3 decades after the war and its relation to military service, combat, and post-traumatic stress disorder. Am J Epidemiol. (2009) 170(3):318–30. 10.1093/aje/kwp146 19564170

[4] Hankin CS Spiro A Miller DR Kazis L. Mental disorders and mental health treatment among U.S. Department of Veterans Affairs outpatients: the Veterans Health Study. Am J Psychiatry. (1999) 156(12):1924–30. 10.1176/ajp.156.12.1924 10588406

[5] Kaiser AP Cook JM Glick DM Moye J. Posttraumatic stress disorder in older adults: a conceptual review. Clin Gerontol. (2019) 42(4):359–76. 10.1080/07317115.2018.1539801 30422749 PMC6666306

[6] Ikin JF Creamer MC Sim MR McKenzie DP. Comorbidity of PTSD and depression in Korean War veterans: prevalence, predictors, and impairment. J Affect Disord. (2010) 125(1):279–86. 10.1016/j.jad.2009.12.005 20071032

[7] Moseley GL Butler DS. Fifteen years of explaining pain: the past, present, and future. J Pain. (2015) 16(9):807–13. 10.1016/j.jpain.2015.05.005 26051220

[8] Wood L Hendrick PA. A systematic review and meta-analysis of pain neuroscience education for chronic low back pain: short-and long-term outcomes of pain and disability. Eur J Pain. (2019) 23(2):234–49. 10.1002/ejp.1314 30178503

[9] Ho EKY Chen L Simic M Ashton-James CE Comachio J Wang DXM Psychological interventions for chronic, non-specific low back pain: systematic review with network meta-analysis. Br Med J. (2022) 376:e067718. 10.1136/bmj-2021-067718 35354560 PMC8965745

[10] Moseley GL Butler DS. The Explain Pain Handbook: Protectometer. Adelaide, Australia: Noigroup Publications (2015).

[11] Moffat AK Apajee J Blanc VTL Westaway K Andrade AQ Ramsay EN Reducing opioid use for chronic non-cancer pain in primary care using an evidence-based, theory-informed, multistrategic, multistakeholder approach: a single-arm time series with segmented regression. BMJ Qual Saf. (2023) 32(11):623–31. 10.1136/bmjqs-2022-015716 37105724 PMC10646855

[12] Cosio D Lin EH. Effects of a pain education program for veterans with chronic, noncancer pain: a pilot study. J Pain Palliat Care Pharmacother. (2013) 27(4):340–9. 10.3109/15360288.2013.846953 24147960

[13] Cashin AG Lee H Wand BM Bagg MK O’Hagan ET Rizzo RRN Mechanisms of education and graded sensorimotor retraining in people with chronic low back pain: a mediation analysis. Pain. (2022) 164(12):2792–800. 10.1097/j.pain.0000000000002978 37366598

[14] Leake HB Mardon A Stanton TR Harvie DS Butler DS Karran EL Key learning statements for persistent pain education: an iterative analysis of consumer, clinician and researcher perspectives and development of public messaging. J Pain. (2022) 23(11):1989–2001. 10.1016/j.jpain.2022.07.008 35934276

[15] Ashar YK Lumley MA Perlis RH Liston C Gunning FM Wager TD. Reattribution to mind-brain processes and recovery from chronic back pain: a secondary analysis of a randomized clinical trial. JAMA Netw Open. (2023) 6(9):e2333846. 10.1001/jamanetworkopen.2023.33846 37768666 PMC10539987

[16] Yu Z Xu W. A meta-analysis and systematic review of the effect of virtual reality technology on users’ learning outcomes. Comput Appl Eng Educ. (2022) 30(5):1470–84. 10.1002/cae.22532

[17] Moseley GL Ryan CG. Making pain education better: historical underpinnings & recent innovations – a discussion paper. PETAL Discussion Papers. (2023). Available at: https://www.petalcollaboration.org/uploads/1/4/4/1/144169171/moseley__ryan_petal_discussion_paper_making_pain_education_better_120923.pdf

[18] Skidmore N Ryan CG Mankelow J Martin D. Acceptability and feasibility of virtual reality to promote health literacy in primary care from the health professional’s view: a qualitative study. Patient Educ Couns. (2024) 123:108179. 10.1016/j.pec.2024.108179 38367303

[19] Levitt HM Bamberg M Creswell JW Frost DM Josselson R Suárez-Orozco C. Journal article reporting standards for qualitative primary, qualitative meta-analytic, and mixed methods research in psychology: the APA Publications and Communications Board Task Force report. Am Psychol. (2018) 73(1):26. 10.1037/amp0000151 29345485

[20] Onwuegbuzie AJ Dickinson WB Leech NL Zoran AG. A qualitative framework for collecting and analyzing data in focus group research. Int J Qual Methods. (2009) 8(3):1–21. 10.1177/160940690900800301

[21] Tashakkori A Teddlie C Teddlie CB. Handbook of Mixed Methods in Social & Behavioral Research. Thousand Oaks, CA: SAGE Publications (2003). p. 792.

[22] Catley MJ O’Connell NE Moseley GL. How good is the neurophysiology of pain questionnaire? A Rasch analysis of psychometric properties. J Pain. (2013) 14(8):818–27. 10.1016/j.jpain.2013.02.008 23651882

[23] Sekhon M Cartwright M Francis JJ. Development of a theory-informed questionnaire to assess the acceptability of healthcare interventions. BMC Health Serv Res. (2022) 22(1):279. 10.1186/s12913-022-07577-3 35232455 PMC8887649

[24] Sekhon M Cartwright M Francis JJ. Acceptability of healthcare interventions: an overview of reviews and development of a theoretical framework. BMC Health Serv Res. (2017) 17(1):88. 10.1186/s12913-017-2031-8 28126032 PMC5267473

[25] Weiner BJ Lewis CC Stanick C Powell BJ Dorsey CN Clary AS Psychometric assessment of three newly developed implementation outcome measures. Implement Sci. (2017) 12(1):108. 10.1186/s13012-017-0635-3 28851459 PMC5576104

[26] Kennedy RS Lane NE Berbaum KS Lilienthal MG. Simulator sickness questionnaire: an enhanced method for quantifying simulator sickness. Int J Aviat Psychol. (1993) 3(3):203–20. 10.1207/s15327108ijap0303_3

[27] Harvie DS Kelly J Kluver J Deen M Spitzer E Coppieters MW. A randomized controlled pilot study examining immediate effects of embodying a virtual reality superhero in people with chronic low back pain. Disabil Rehabil Assist Technol. (2022):1–8. 10.1080/17483107.2022.2129846 36256688

[28] Botvinick M Cohen J. Rubber hands ‘feel’ touch that eyes see. Nature. (1998) 391(6669):756. 10.1038/35784 9486643

[29] Makransky G Lilleholt L Aaby A. Development and validation of the multimodal presence scale for virtual reality environments: a confirmatory factor analysis and item response theory approach. Comput Human Behav. (2017) 72:276–85. 10.1016/j.chb.2017.02.066

[30] Morgan DL Krueger RA King JA. Developing Questions for Focus Groups. Thousand Oaks, CA: SAGE (1998). p. 130.

[31] Dilgul M Hickling LM Antonie D Priebe S Bird VJ. Virtual reality group therapy for the treatment of depression: a qualitative study on stakeholder perspectives. Front Virtual Real. (2021) 1:609545. 10.3389/frvir.2020.609545

[32] Nadarzynski T Miles O Cowie A Ridge D. Acceptability of artificial intelligence (AI)-led chatbot services in healthcare: a mixed-methods study. Digital Health. (2019) 5:2055207619871808. 10.1177/2055207619871808 31467682 PMC6704417

[33] Ghiara V. Disambiguating the role of paradigms in mixed methods research. J Mix Methods Res. (2020) 14(1):11–25. 10.1177/1558689818819928

[34] Hesse-Biber SN Johnson RB. The Oxford Handbook of Multimethod and Mixed Methods Research Inquiry. Oxford, United States: Oxford University Press, Incorporated (2015). Available at: http://ebookcentral.proquest.com/lib/unisa/detail.action?docID=2044599 (Accessed April 17, 2023).

[35] Panhwar DAH Ansari D Shah A. Post-positivism: an effective paradigm for social and educational research. Int Res J Arts Hum. (2017) 45:253–60.

[36] Gale NK Heath G Cameron E Rashid S Redwood S. Using the framework method for the analysis of qualitative data in multi-disciplinary health research. BMC Med Res Methodol. (2013) 13(1):117. 10.1186/1471-2288-13-117 24047204 PMC3848812

[37] Green J Caracelli V Graham W. Toward a conceptual framework for mixed-method evaluation designs. Educ Eval Policy Anal. (1989) 11:255–74. 10.3102/01623737011003255

[38] Royal Commission welcomes AIHW report containing updated suicide data | Royal Commission into Defence and Veteran Suicide (2022). Available at: https://defenceveteransuicide.royalcommission.gov.au/news-and-media/media-releases/royal-commission-welcomes-aihw-report-containing-updated-suicide-data (Accessed July 10, 2024).

[39] Watson JA Ryan CG Cooper L Ellington D Whittle R Lavender M Pain neuroscience education for adults with chronic musculoskeletal pain: a mixed-methods systematic review and meta-analysis. J Pain. (2019) 20(10):1140.e1–e22. 10.1016/j.jpain.2019.02.011 30831273

[40] Robinson V King R Ryan CG Martin DJ. A qualitative exploration of people’s experiences of pain neurophysiological education for chronic pain: the importance of relevance for the individual. Man Ther. (2016) 22:56–61. 10.1016/j.math.2015.10.001 26511524

[41] Lorimer Moseley G Leake HB Beetsma AJ Watson JA Butler DS van der Mee A Teaching patients about pain: the emergence of pain science education, its learning frameworks and delivery strategies. J Pain. (2024) 25(5):104425. 10.1016/j.jpain.2023.11.008 37984510

[42] Wilson MV Braithwaite FA Arnold JB Stanton TR. Real-world implementation of pain science education and barriers to use in private practice physiotherapy settings: an Australia-wide cross-sectional survey. Pain. (2025). 10.1097/j.pain.0000000000003521 39869479

[43] Bruner JS. Beyond the information given: studies in the psychology of knowing. In: Anglin JM, editor. Beyond the Information Given: Studies in the Psychology of Knowing. New York, NY: Norton Agency Titles (1973). p. xxiv, 502.

[44] Kothgassner OD Goreis A Kafka JX Van Eickels RL Plener PL Felnhofer A. Virtual reality exposure therapy for posttraumatic stress disorder (PTSD): a meta-analysis. Eur J Psychotraumatol. (2019) 10(1):1654782. 10.1080/20008198.2019.1654782 31489138 PMC6713125

[45] Moseley GL. Whole of community pain education for back pain. Why does first-line care get almost no attention and what exactly are we waiting for? Br J Sports Med. (2019) 53(10):588–9. 10.1136/bjsports-2018-099567 29982226

[46] Vallerand AH Cosler P Henningfield JE Galassini P. Pain management strategies and lessons from the military: a narrative review. Pain Res Manag. (2015) 20(5):261–8. 10.1155/2015/196025 26448972 PMC4596634

[47] Lee H Lamb SE Bagg MK Toomey E Cashin AG Moseley GL. Reproducible and replicable pain research: a critical review. Pain. (2018) 159(9):1683–9. 10.1097/j.pain.0000000000001254 29697535

[48] Karran EL Fryer CE Middleton JW Moseley GL. Exploring the social determinants of health outcomes for adults with low back pain or spinal cord injury and persistent pain: a mixed methods study. J Pain. (2022) 23(9):1461–79. 10.1016/j.jpain.2022.04.001 35429673

[49] Karran EL Grant AR Moseley GL. Low back pain and the social determinants of health: a systematic review and narrative synthesis. Pain. (2020) 161(11):2476–93. 10.1097/j.pain.0000000000001944 32910100

[50] Karran EL Cashin AG Barker T Boyd MA Chiarotto A Dewidar O Using PROGRESS-plus to identify current approaches to the collection and reporting of equity-relevant data: a scoping review. J Clin Epidemiol. (2023) 163:70–8. 10.1016/j.jclinepi.2023.09.017 37802205

[51] Mardon AK Chalmers KJ Heathcote LC Curtis LA Freedman L Malani R “I wish I knew then what I know now” - pain science education concepts important for female persistent pelvic pain: a reflexive thematic analysis. Pain. (2024) 165(9):1990–2001. 10.1097/j.pain.0000000000003205 38452219

[52] Moore E Braithwaite FA Stanton TR Bellan V Moseley GL Berryman C. What do I need to know? Essential educational concepts for complex regional pain syndrome. Eur J Pain. (2022) 26(7):1481–98. 10.1002/ejp.1976 35598314 PMC9542775

[53] Leake HB Heathcote LC Simons LE Stinson J Kamper SJ Williams CM Talking to teens about pain: a modified Delphi study of adolescent pain science education. Can J Pain. (2019) 3(1):200–8. 10.1080/24740527.2019.1682934 35005410 PMC8730612

